# Global Health Partnerships: A Perspective of Eye Health Initiatives from the Uganda–United Kingdom Engagements and the Global Health Agenda

**DOI:** 10.5334/aogh.4769

**Published:** 2025-08-20

**Authors:** Primrose Magala, Innocent Ayesiga, Ian Yeung, Samuel Mbayo, Moses W. Mulimira, Sheba G. Nakacubo

**Affiliations:** 1 Moorfields Eye Hospital, NHS, London, UK; 2 Department of Research, Eye Health Africa, Kampala, Uganda; 3 Department of Community Health, Mbarara University of Science and Technology, Uganda; 4 Department of Research, Global Health Partnerships (formerly THET), Kampala, Uganda

**Keywords:** global health partnerships, Uganda–UK, eye health, diaspora health professionals, global health agenda

## Introduction

**Figure 1:**
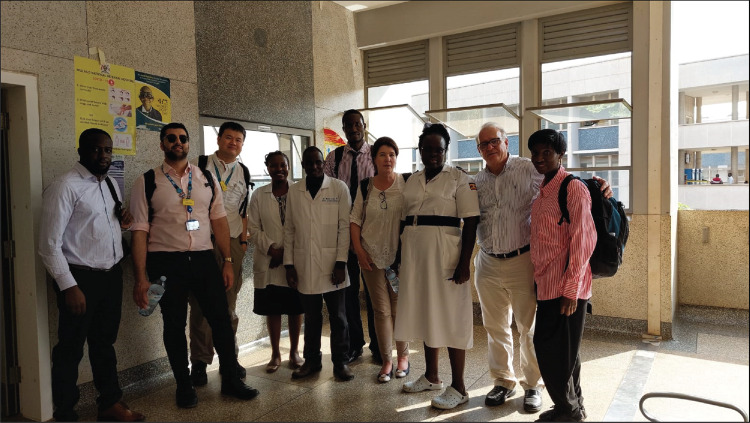
The photo shows the UK and Brazil specialists with some local healthcare workers, such as resident ophthalmologists and ophthalmic nurses, at Mulago National Referral Hospital in Uganda during the 2023 visit.

During our initial visits to Uganda in early 2017, we noticed that most older people had significant visual impairment due to cataracts. Many of these cataract patients never bothered about receiving intervention, and yet this affected their daily activities. This experience sparked the passion for engaging multiple eye health experts from Moorfields Eye Hospital, UK, to come to Uganda and offer eye care medical services through partnering with local health facilities. Eye Health Africa (EHA) CIC was birthed through these partnerships to improve eye health across African countries through North–South partnerships and international expert exchanges to foster learning and enhanced capacity development. EHA has continuously mobilized UK eye specialists to come to Uganda annually to extend healthcare services to the people as demonstrated in Figure 1.

Global health partnerships have become a crucial aspect of the global health agenda, recognizing the interconnectedness of health systems and the need for collaborative efforts to address health inequalities. The Sustainable Development Goals (SDGs), particularly Goal 3 (ensure healthy lives and promote well-being for all ages), emphasize the importance of partnerships in achieving global health goals. Eye health is essential to global health, with an estimated 1.6 billion people worldwide living with vision impairment or blindness. Of these, 89% reside in low- and middle-income countries, where access to eye care services is often limited [1]. The World Health Organization (WHO) estimates that 80% of vision impairment can be prevented or cured, highlighting the need for effective partnerships to address this significant health burden. Uganda, like many sub-Saharan African countries, faces significant eye health challenges [1]. According to the Uganda National Eye Health Survey, over 1.4 million people in Uganda live with vision impairment or blindness, with cataracts, refractive errors, and glaucoma being the leading causes [2]. The country has only approximately 60 ophthalmologists serving a population of over 45 million, emphasizing the need for capacity building and partnerships to strengthen eye health services. Significantly, the Uganda–United Kingdom (UK) health partnerships have a long history, dating back to the early 20th century. These partnerships have focused on various health areas, including eye health, with collaborations between Ugandan and UK institutions, organizations, and individuals. The UK, primarily through its initiatives from the Foreign and Commonwealth Development Office (FCDO), has supported global health initiatives, including the WHO’s Vision 2020 program, which aims to eliminate avoidable blindness by 2020. Global health partnerships, particularly between Uganda and the UK, are critical in addressing eye health disparities. By leveraging resources, expertise, and knowledge, these partnerships can strengthen eye health systems and services, build capacity for eye health professionals, and improve access to eye care services, particularly in rural and underserved areas. Additionally, these partnerships can support research and innovation in eye health and advocate for eye health policy and programming. This opinion piece explores the Uganda–UK engagements in eye health, highlighting successes, challenges, and opportunities for future collaborations to address the significant eye health burden in Uganda, Sub-Saharan Africa, and globally.

## Stakeholder Engagements

USAID emphasizes the importance of a shared vision between partners*: “The most effective partnerships are those in which risks, responsibilities, and rewards are shared, and which address core interests of all parties involved*” [3]. Stakeholder engagement has been a cornerstone of Uganda–UK collaborations in eye health. These engagements have brought together government institutions, non-governmental organizations, healthcare professionals, academic institutions, and community leaders to ensure that interventions are contextually relevant and sustainable. A notable example is the collaboration between Moorfields Eye Hospital and Ugandan health institutions, such as Mulago National Referral Hospital and Hoima Regional Referral Hospital [4]. Through this partnership, specialized training programs for ophthalmologists and eye care workers have been implemented, significantly improving the capacity of Uganda’s health workforce. Similarly, the involvement of community health workers and diaspora health professionals in outreach programs has enhanced the delivery of eye care services to remote and underserved communities. Notably, diaspora healthcare professionals are healthcare workers who have trained in one healthcare system before immigrating to another and contribute significantly to health both in the UK and overseas, bringing a wealth of expertise and skills. Diaspora health professionals are powerhouses of creativity and innovation in their countries of residence and origin. They have knowledge, skills, and global connections crucial to delivering healthcare in under-resourced areas and on much larger scales. This dual insight of healthcare in the UK and overseas can be a conduit for bilateral learning and growth, improving patient care and system efficiencies.

## Impact

The Uganda–UK partnerships have yielded significant impacts on eye health in Uganda. For example, there has been increased capacity building and training for ophthalmologists, optometrists, and other eye care professionals. Significantly, over 40 ophthalmology residents have interacted with over 30 eye health specialists from the UK, which has led to the exchange of knowledge and skills [4]. Moreover, surgical skills have also been shared in critical areas, such as oculoplastics, vitreoretinal, and retinopathy of prematurity. These have collectively improved service access and increased eye care awareness through over ten public awareness campaigns. Notably, the discussions from these engagements have contributed to the formulation of the Uganda National Eye Health Policy with some stakeholder meetings, such as the Director General of Clinical Services and the commissioner for eye health at the Ugandan Ministry of Health.

## Discussion

Despite these achievements, Uganda’s eye health sector still faces challenges, such as limited funding, inadequate infrastructure, and a shortage of trained personnel. Furthermore, the reliance on external financing raises concerns about the sustainability of some initiatives. Addressing these issues requires a multifaceted approach that includes strengthening local health systems, increasing domestic funding, and fostering more equitable partnerships. Some opportunities for future collaboration include scaling up the existing models of community-based eye care, leveraging technological models like telemedicine to expand access to specialized care, and strengthening research collaborations to generate evidence for eye care policy and practice. Additionally, encouraging private-sector engagement and investments in eye care initiatives must be prioritized to enhance the provision of services to multiple underserved communities.

## Conclusion

The Uganda–UK partnerships in eye health represent the potential of global health collaborations to address significant health challenges. By building on past successes and addressing existing gaps, these partnerships can continue to improve eye health outcomes in Uganda and serve as a model for other low- and middle-income countries. Moving forward, we recommend strengthening local capacity through increased investment in training and infrastructure, fostering equitable partnerships that prioritize mutual learning and shared responsibility, and advocating for increased domestic funding for eye health programs. Additionally, we suggest the expansion of innovative technologies to improve service delivery. These innovative technologies must be designed based on the local community requirements within low- and middle-income countries. Finally, we recommend enhancing monitoring and evaluation mechanisms to ensure the sustainability and impact of multiple eye health projects.

## Figures and Tables

**Table:** 

FCDO	Foreign and Commonwealth Development Office
SDGs	Sustainable Development Goals
WHO	World Health Organization
